# Gut microbial carcinogen metabolism: another avenue to cancer

**DOI:** 10.1038/s41392-024-02015-8

**Published:** 2024-10-28

**Authors:** Florian R. Greten, Melek C. Arkan

**Affiliations:** 1 Institute for Tumor Biology and Experimental Therapy, Georg-Speyer-Haus, Frankfurt/Main, Germany; 2 Frankfurt Cancer Institute, Goethe University Frankfurt, Frankfurt/Main, Germany; 3 German Cancer Consortium (DKTK) and German Cancer Research Center (DKFZ), Heidelberg, Germany

**Keywords:** Urological cancer, Cell biology

A recent study published in *Nature* by Roje, Zhang and colleagues highlights the emergent role gut microbiota play in processing environmental carcinogens and raises its potential as a target for reducing cancer risk in humans.^1^ This study fills yet another piece into the giant jigsaw puzzle that illustrates the central role of the dynamic structure and function of the intestinal microbiome in cancer pathogenesis and therapy efficacy.

Growing amount of evidence indicates that the human microbiota play a key role in both the initiation and progression of cancer. While the exact molecular pathways by which microbes contribute to cancer are not yet fully understood, it has been shown that *i*. bacterial products such as LPS can initiate tumor promoting inflammatory conditions, *ii*. certain bacteria can migrate from the gut to distant tissues, and *iii*. some bacteria can produce genotoxins like colibactin, which can trigger tumor formation.^2^ Yet, another crucial mechanism in cancer development is exposure to environmental pollutants, which, once inside the body, are often metabolized further by enzymes such as cytochrome P450, that are well-documented for their ability to either activate or deactivate a wide range of carcinogens.

**Fig. 1:**
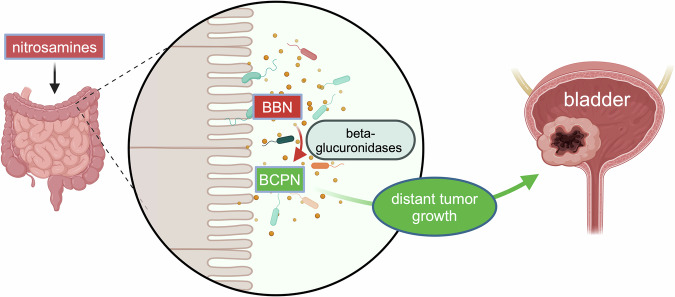
The intestinal microbiome generates carcinogens. Nitrosamines, that can be ingested with food, are metabolized by the intestinal microbiota. Certain bacterial strains have the metabolic capacity to lead to the formation of carcinogenic nitrosamines such as *N*-butyl-*N*-(3-carboxypropyl)-nitrosamine (BCPN), the oxidation product of *N*-butyl-*N*-(4-hydroxybutyl)-nitrosamine (BBN). BCPN can then induce DNA damage and initiate tumors in distant organs such as bladder. Created with BioRender.com

The study by Roje, Zhang and their colleagues demonstrates that gut microbiota directly affect toxicokinetics of nitrosamines, the organic compunds that are carcinogenic, thereby impacting the development and severity of nitrosamine-induced urinary bladder cancer in mice. *N*-butyl-*N*-(3-carboxypropyl)-nitrosamine (BCPN), the oxidation product of the nitrosamine compound *N*-butyl-*N*-(4-hydroxybutyl)-nitrosamine (BBN), induces tumorigenesis through DNA adduct formation in the urothelium. Interestingly, antibiotic treatment nearly completely ablated tumor formation in a pre-clinical mouse model of BBN-induced bladder cancer following reduction of the gut bacterial load leading to decreased urinary BCPN elimination and urothelial BCPN exposure. While the levels of BCPN in liver, kidney and plasma remained indifferent between antibiotic-treated and untreated control animals, BCPN levels were significantly reduced in the large intestine upon depletion of intestinal microbiota. This was explained by differences in intestinal deconjugation of glucuronidated BBN by gut β-glucuronidases and oxidation of liberated BBN to BCPN in a microbiome-dependent fashion (Fig. 1).

The authors were able to identify four putative BBN-metabolizing bacterial genera *(Escherichia, Lactobacillus*, *Corynebacterium* and *Staphylococcus)*, indicating that only a distinct subpopulation of the gut microbiota could confer this effect. Importantly, these findings were not limited to mouse microbial communities, but BBN-converting community members could be identified also in human gut microbiota. Despite the distinct taxonomic compositional differences between mouse and human gut microbiome, *Escherichia* was the only one overlapping. In an elegant in vivo approach, the capacity of these bacterial strains to metabolize BBN and convert to BCPN was confirmed when combinations of bacterial strains were used to recolonize the intestine of gnotobiotic mice and strain-dependent intestinal BCPN production was observed with pronounced inter-individual differences in metabolism of this environmental pollutant. Finally, the relevance of these findings were extended to other related nitrosamine carcinogens including (*N*-ethyl-*N*-(4-hydroxybutyl)-nitrosamine (EHBN), *N*-nitrosodibutylamine (DBN) and *N*-propyl-*N*-butyl-nitrosamine (PBN) thus providing direct evidence that gut microbiota-dependent deconjugation and oxidation reactions were able to alter the toxicokinetics of also these nitrosamine carcinogens, which indicated a general effect of bacterial communities to drive chemically-induced tumorigenesis.

Curiously, levels of the active carcinogen BCPN were reduced in the intestine as well as in bladder tissue and urine in antibiotic-treated mice while plasma or liver BCPN levels remained unchanged raising the question about the actual kinetics of BCPN transfer from intestine to bladder. Since antibiotic treatment led to a massive reduction in tumor load in carcinogen-treated mice, one cannot rule out that the comparison of non-transformed bladder tissue to maligant tumor may contribute to the differences detected in BCPN levels. Thus, the lack of other tumor promoting microbiome-dependent mechanisms or secondary changes in the intestine such as fungal outgrowth, which can commonly occur upon antibiotic-induced bacteria depletion, could contribute to the marked difference in tumor incidence.

The current study adds another layer to previous studies providing significant insight into the intricate connection between host-microbiome interactions, and the dynamics of bacterial biotransformation reactions in determining toxicokinetics of carcinogens. By demonstrating the existence of inter-species interactions for converting BBN to BCPN, and inter-individual differences in BBN metabolism, this research highlights the necessity of considering microbial community structure and function when studying environmental carcinogens in cancer development.

The human body is continuously exposed to xenobiotics (environmental pollutants, carcinogens, drugs, pesticides, fumigants, disinfectants, and food additives) that reach the gut microbiome directly or indirectly after being metabolized in the liver.^3^ Gut function via absorption, distribution, biotransformation, and excretion affects xenobiotic disposition. Importantly, environmental xenobiotics have been suggested to have themselves a profound impact on gut microbiota by shaping their composition and boosting the selection of strains with xenobiotic degrading capacity.^4^ Yet, microbial strains that seem to be functionally redundant within a metabolic niche can have diverse impacts outside the niche.^5^ The findings in this study confirm that context-specific community structure may be the major determinant for bacterial deconjugation and oxidation reactions that then determines toxicokinetics of carcinogenic nitrosamines. However, this brings us back to square one that understanding the community function is way too complex and cannot be achieved by a single species or strain. This is especially becoming apparent when taking a distinct metabolic niche out of one individuals’ context and trying to transfer it into another environment. These data raise concerns especially for therapeutic approaches such as fecal transplantations to treat non-tumorigenic diseases that may unintentionally lead to expansion of carcinogen metabolizing strains and thus unwanted increased risk of cancer development. Thus, it is important to illuminate microbe-microbe interactions first, before we can mechanistically understand microbiome drug metabolism at a community-level and design targeted-strategies to manipulate individuals’ microbiota to beneficially modify contextual host-microbiome metabolic interactions in cancer.

Nevertheless, the findings by Roje and Zhang build an exciting basis for future investigations regarding the capacity of microbial communities influencing carcinogen metabolism and underscore that we are still far away from a comprehensive understanding of the multitude of functions our gut microbiome is responsible for as well the potential of microbiome-based therapeutic interventions for cancer prevention it offers.
